# Direct and Indirect Effects of Child Maltreatment on Suicidal Ideation among Chinese Left-Behind Children: Does Gender Make a Difference?

**DOI:** 10.3390/bs12110464

**Published:** 2022-11-20

**Authors:** Xiaoyan Fan, Xiangping Li

**Affiliations:** School of Social Development, East China Normal University, Shanghai 200241, China

**Keywords:** maltreatment, suicidal ideation, self-efficacy, gender, left-behind children

## Abstract

Previous studies indicate that maltreatment is related to children’s suicidal ideation, but the indirect mechanisms of left-behind children have been rarely investigated in the Chinese context. On the basis of a left-behind children sample (N = 1355; 57.1% females), this study aims to investigate the direct and indirect effects of maltreatment on suicidal ideation among Chinese left-behind children. The results of structural equation modeling (SEM) demonstrate that child maltreatment not only directly affects the suicidal ideation of left-behind children, but can also indirectly affect their suicidal ideation through the partially mediating role of self-efficacy. Moreover, a significant gender difference was found in the direct effect of maltreatment on suicidal ideation, with females experiencing stronger influence than males. Findings suggest that the effect of maltreatment on the suicidal ideation of left-behind children is mediated by self-efficacy and moderated by gender. Findings also highlight intervention directions for risk behaviors among left-behind children.

## 1. Introduction

As a global public health concern [1], child maltreatment refers to behaviors that pose a substantial risk of physical or emotional harm to children. Child maltreatment has five categories of abuse and neglect, including physical abuse, emotional abuse, sexual abuse, emotional neglect, and physical neglect [2,3]. According to the 2020 report released by the WHO and UNICEF, approximately 50% of children aged 2–17 years experience some form of maltreatment each year worldwide. Nearly 300 million children aged 2–4 years regularly suffer from violent discipline by their caregivers. Maltreatment not only negatively impacts children’s mental health and may even have a long-term impact on their survival and development [4,5], but it also places a significant financial burden on families and the state [6]. Thus, the prevalence and serious impact of child maltreatment require urgent attention from governments and researchers.

Migrant children in cities lack access to high-quality education and social security due to restrictions in the household registration system in Mainland China. Thus, the children of migrant workers are left behind in rural areas. The left-behind children are separated from their parents for an extended period of time, leaving them in an environment devoid of parental involvement and family protection [7]. Parental absence reduces parental protection and family support, and left-behind children are more likely to suffer from various negative experiences [8]. Compared with non-left-behind children, the left-behind children are at a higher risk of child maltreatment. Due to parental absence, malnutrition, material deprivation, and lack of parent–child interaction and parental care, left-behind children are more likely to experience physical and emotional abuse and neglect from their parents [9]. Meanwhile, most left-behind children are cared for by their grandparents, which can result in physical and emotional abuse due to survival conditions, caregiving pressure, and parenting styles [10]. In addition, left-behind children are in a relatively vulnerable position in rural areas, and they may also suffer physical, emotional and even sexual abuse from other caregivers, teachers, and community members [11].

Previous studies have shown that child maltreatment tend to have long-lasting effects throughout an individual’s life [12]. Child maltreatment not only affects children’s mental health (e.g., character defects, inferiority, self-doubt), but also has serious consequences for their behaviors (e.g., aggression, suicidal ideation, and behaviors) [4,13,14]. The WHO’s report in 2021 indicated that maltreatment is a significant risk factor for suicide among adolescents aged 15–19 years. In addition, relevant empirical studies have confirmed that child maltreatment is a significant predictor of suicidal ideation and suicidal behavior in children. Therefore, effective measures and interventions must be taken to reduce child maltreatment and individuals’ suicidal ideation and behavior. Under this background, this study analyzes the current situation of maltreatment experiences and suicidal ideation of left-behind children and explores how child maltreatment directly and indirectly affects left-behind children’s suicidal ideation. This study also investigates whether significant gender differences exist in the relationship between child maltreatment and suicidal ideation in the Chinese context. The findings would provide implications on policies and interventions to prevent child maltreatment and suicidal ideation among left-behind children.

## 2. Literature Review

### 2.1. Child Maltreatment and Suicidal Ideation

The relationship between child maltreatment and suicidal ideation has been investigated in previous theories and research articles. At the theoretical level, according to the integrated motivational volitional model, early life adversity is an unequivocal suicidal risk factor. Negative life events experienced at any stage in the life course are the pre-motivational factors that affect individual’s suicide risk [15]. Accordingly, maltreatment as negative life events experienced in childhood is a risk factor for suicidal ideation and intention in people’s life courses.

Empirical studies also verified the association between child maltreatment and children’s suicidal ideation. Studies show that all forms of maltreatment make children at risk for suicidal ideation and behavior. In Australia, the suicide rate for maltreated children was 10.7–13.0 times higher than for non-maltreated children [16]. A study in China also confirmed that all forms of child maltreatment have been linked to a higher likelihood of suicide [17]. In addition, the effects of different forms of maltreatment on children’s suicidal ideation may vary across nations and groups [18,19]. Data from a national longitudinal study in the United States indicate that sexual, emotional, and physical abuse may confer the onset or exacerbation of suicidal ideation, but neglect cannot increase the risk for suicidal ideation [20]. Contrastingly, research in Brazil demonstrated contradictory findings that emotional neglect is directly associated with suicidal ideation; however, physical neglect and sexual abuse are not [21]. In a German network analysis, sexual abuse is the only sub-type of abuse directly related to suicidal ideation [22], whereas a recent meta-analysis shows that physical abuse has the strongest effect on suicidal ideation [23]. Compared with non-left-behind children, the left-behind children reported a high risk of suicidal ideation if they ever suffered any type of maltreatment [24]. For left-behind children, emotional neglect and physical abuse are two risk factors of suicidal ideation, which has been illustrated by a survey in Hunan investigating 1167 students [25]. Furthermore, Mossige et al. suggested that the effect maltreatment had on suicidal ideation also depended on the period of experiencing maltreatment. Maltreatment experienced during childhood tend to have a greater impact on children’s suicidal ideation than maltreatment experienced at a later age [26]. Thus, we propose the following hypothesis:

**H1:**  *Child maltreatment is positively correlated to left-behind children’s suicidal ideation.*

### 2.2. Self-Efficacy as Mediator

Self-efficacy was defined as “an individual’s beliefs about his/her own capabilities to organize and execute courses of action required for accomplishing a specific task [27]”, suggesting that self-efficacy can affect individuals’ beliefs about their abilities to perform a specific task. According to the developmental psychopathology perspective [28], traumatic childhood experiences contribute to dysfunctional and maladaptive behaviors by distorting the self-system [29]. As a personal conviction in one’s aptitude and capacity to achieve given attainments [30], self-efficacy is a critical component of the individual’s self-system. Due to the immaturity of physical and mental development, exposure to maltreatment may impede stage-salient developmental tasks and disrupt a child’s self-system [31]. With reduced positive self-perception and increased negative self-concept [32,33], maltreated individuals exhibit a range of psychopathological disorders and even develop suicidal ideation and behavior [34,35]. Thus, self-efficacy can mediate the relationship between child maltreatment and suicidal ideation.

Empirical studies have confirmed the impact of child maltreatment on their self-efficacy. Children’s self-efficacy is affected by their living environment and life experiences [36]. Living in an abusive situation can shape or alter individuals’ inner and outer perceptions, which may relate to deficits in social cognition and self-efficacy [37,38]. Extensive empirical research identified a low perceived self-efficacy as a potentially detrimental outcome of early maltreatment exposures [39,40]. Using longitudinal data from a sample of 739 adolescent girls and boys aged between 14 and 18 years in Canada, Marie-Pier’s research found a strong correlation between child sexual abuse and self-efficacy [41]. Moreover, emotional abuse and neglect were related to lower levels of resilience [42].

Empirical research demonstrated that a high sense of self-efficacy can be considered an essential protector to individuals’ mental health and behaviors [43]. Individuals with high self-efficacy typically believe in their ability to cope with stressful situations, which can keep them from dwelling on negative emotions, particularly suicidal ideation [38]. Using cross-sectional data with 3836 adolescents, a South Carolina survey revealed that low levels of emotional self-efficacy would be significantly associated with higher levels of suicidal ideation and suicidal attempts [44]. A study carried out in Malaysia also found that self-efficacy had a significant negative correlation with suicidal ideation among adolescents [38]. On the basis of the theoretical and practical evidence, we propose the following hypothesis:

**H2:**  *Self-efficacy mediates the association between child maltreatment and left-behind children* *’s suicidal ideation.*

### 2.3. Gender Differences

Although gender differences in child maltreatment and suicidal ideation have been investigated [45], some controversies exist. For example, compared with males, female victims in the US have a higher prevalence of suicidal ideation [46]. Similarly, findings from a cross-sectional study in the United Kingdom support that sexual abuse more commonly leads to suicidal ideation in females than in males [47]. However, a study conducted in Africa uncovered that the association between maltreatment and suicidal ideation is far more significant among boys rather than girls [48]. Despite a “gender paradox” relationship between maltreatment exposures and suicidal behaviors due to the sex-disaggregated biological mechanisms [48], other studies also claimed that different gender roles do not weaken or exacerbate the effect of maltreatment on suicidal ideation. For example, a systematic review revealed that gender does not moderate the association between maltreatment and suicidal attempts of children [23]. Under this controversial background, this study aims to further examine the gender differences in the Chinese context to understand of the relations among these variables.

**H3:**  *The effect of child maltreatment on left-behind children* *’s suicidal ideation may differ across gender.*

### 2.4. The Knowledge Gaps and the Framework

**Figure 1:**
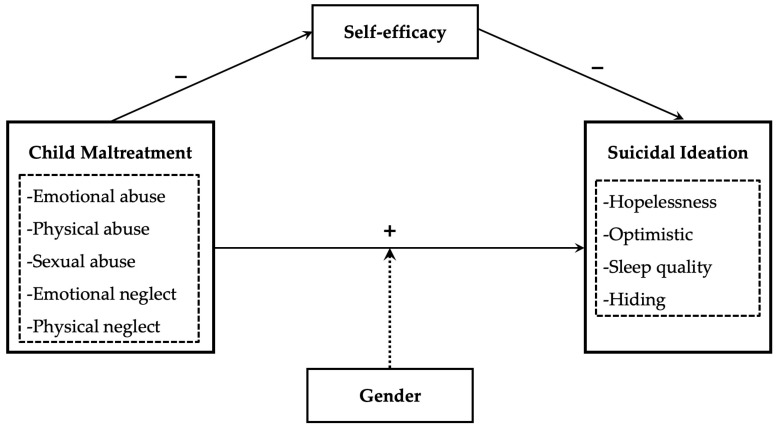
Theoretical framework.

Several knowledge gaps exist regarding the direct and indirect association between child maltreatment and left-behind children’s suicidal ideation. First, although some studies have verified the direct effect of maltreatment on suicidal ideation in the general population of children and adolescence, empirical studies on the vulnerable group of left-behind children in the Chinese context are relatively limited. Second, fewer studies verified the mediating effect of self-efficacy on the relationship between maltreatment and left-behind children’s suicidal ideation. Thus, the indirect mechanism warrants further exploration. Third, some controversies exist regarding gender differences. Some studies suggested that no significant gender difference exists in the relationship between maltreatment and suicidal ideation. However, some studies reveled that gender may moderate the effect, but whether the effect is greater for males or females remains controversial. Thus, this study must further analyze the possibility of gender differences of left-behind children in the Chinese context. This study aims to fill the knowledge gaps by investigating how child maltreatment directly and indirectly affects left-behind children’s suicidal ideation. The theoretical framework is present in Figure 1.

## 3. Methods

### 3.1. Data

The data were collected from a project of children’s health and development conducted in Jiangsu Province. Using multi-stage cluster sampling method, eight middle and high schools were selected as the research sites for the questionnaire survey. Before conducting the questionnaire survey, the research aims and research questions were explained to the teachers and students. Meanwhile, we sent the informed consent forms to the students and their parents. If they agreed and signed the forms, we distributed the questionnaires to students. The survey was administered in the classroom during the students’ extracurricular activity time. It took about approximately 30–40 min. The researchers checked all the questionnaires when they were handed in. Ultimately, we collected 1355 valid samples of left-behind children. In this study, we strictly followed research ethics, and all the materials were submitted for review and approval by the research ethics committee of the authors’ university.

### 3.2. Measurement

#### 3.2.1. Maltreatment

The Childhood Trauma Questionnaire-Short Form (CTQ-SF) is adopted to evaluate child maltreatment [49]. The CTQ-SF evaluates children’s experiences of abuse and neglect, including emotional abuse, physical abuse, sexual abuse, emotional neglect, and physical neglect. It has 28 items, each of which is scored on a five-point Likert scale (never = 1 to always = 5). We calculate the mean of five dimensions to indicate the maltreatment status of left-behind children. Higher scores indicate higher level of maltreatment experiences. The scale has good reliability and validity and has been widely used in previous studies across different cultures. In the current study, the Cronbach’s alpha is 0.904.

#### 3.2.2. Suicidal Ideation

The self-rating idea of suicidal scale (SIOSS) consists of 26 items measuring children’s suicidal ideation. It is developed by Xia [50] and is applicable in the Chinese context. The scale includes four dimensions to reflect the suicidal ideation: hopelessness, optimistic, sleep quality and hiding. Items are graded on a two-point scale with Yes (1) or No (0). We sum up the mean scores of four dimensions to assess left-behind children’s suicidal ideation. The four dimensions are regarded as the observed variables of suicidal ideation in this study. Higher scores indicate that the left-behind children have a higher level of suicidal ideation in that dimension. The Chinese version of this scale was widely used in many related Chinese studies and proved to have good validity and reliability. The Cronbach’s alpha is 0.859 in the current study.

#### 3.2.3. Self-Efficacy

The Chinese version of General Self-Efficacy Scale (CGSES) is adopted to measure the participant’s self-efficacy [51]. The scale comprises ten items and is graded on a five-point scale, from “1 = completely disagree” to “5 = absolutely agree”. The sum of each items yields a self-efficacy total score, which ranges from 10 (poor self-efficacy) to 50 (high self-efficacy). We calculate the mean to indicate the self-efficacy of left-behind children. The CGSES is one of the most widely used scales to assess self-efficacy, and substantial research has supported its validity, reliability, and applicability across cultures. In our study, the Cronbach’s alpha value is 0.868.

#### 3.2.4. Socio–Demographic Variables

Some variables are controlled in this study: Gender (male = 1, female = 2), age, household registration (rural = 1, urban = 2), only child (no = 0, yes = 1), and single parent families (no = 0, yes = 1).

### 3.3. Data Analysis

The data analysis is divided into two parts: descriptive statistics and structural equation modeling (SEM) analysis. In the descriptive statistics part, we mainly use SPSS 25.0 to calculate the frequency and percentage to describe the main characteristics of left-behind children, and we also analyze the correlations among the core variables with Pearson correlations. In the SEM part, we mainly used Amos 25.0 to investigate the direct and indirect effect. First, the validation of the measurement model was tested through confirmatory factor analysis (CFA). Second, we used path analysis to verify the direct and mediating effect. Third, multi-group analysis was employed to test the gender differences on the relationship between maltreatment and suicidal ideation. In the SEM, x^2^, CFI, GFI, and RMSEA are used to determine the fitness of the model. The p-value of the chi-square test was greater than 0.05, which indicates that the data fit well with the model. The GFI and CFI are both higher than 0.9, and the RMSEA is less than 0.08, indicating that the data fits the model well.

## 4. Results

### 4.1. Descriptive Statistics

Table 1 shows the descriptive statistics results of the socio–demographic variables, including frequency, percentage, and means. In the 1355 sample, there are 581 males (42.9%) and 774 females (57.1%). The mean age of the participants is 14.99 years (S.D. = 1.826) in the sample. In terms of the household registration type, 44.3% of the left-behind children have rural household registration, while 55.7% have urban household registration. In terms of whether they are only children and from single-parent families, 43.8% are only children, and 13.8% are from single-parent families.

### 4.2. Correlations among the Key Variables

Pearson correlations (Table 2) are conducted to test the correlations among the key variables in this study. We can see that all the key variables are correlated with each other. Maltreatment is positively correlated with left-behind children’s suicidal ideation (r = 0.565, *p* < 0.01) and negatively related to self-efficacy (r = −0.352, *p* < 0.01). Self-efficacy is negatively correlated with the suicidal ideation of left-behind children (r = −0.356, *p* < 0.01).

### 4.3. Measurement Model

The results indicate that the measurement model fits well with the data. χ^2^ = 81.347(df = 31), *p* < 0.001, CFI = 0.973 > 0.9, GFI = 0.973 > 0.9, RMSEA = 0.051 < 0.08; the three indicators of CFI, GFI and RMSEA suggest that the data has a good fit to the model.

In this study, the two latent variables of maltreatment and suicidal ideation are composed of the corresponding observed variables. Results demonstrate that the standardized factor loadings of all observed variables of maltreatment and suicidal ideation range from 0.431 to 0.820 (Table 3), which indicate that all observed variables could better reflect the constructs of the latent variables.

### 4.4. Structural Model

Results of SEM suggest that the structural model fits well to the data. χ^2^ = 140.8 (df = 59), *p* < 0.001, CFI = 0.960 > 0.9, GFI = 0.968 > 0.9, RMSEA = 0.047 < 0.08; the CFI, GFI and RMSEA suggest that the data has a good fit to the structural model. The findings also indicate that 63.6% of the suicidal ideation in left-behind children was explained by the overall model.

**Figure 2:**
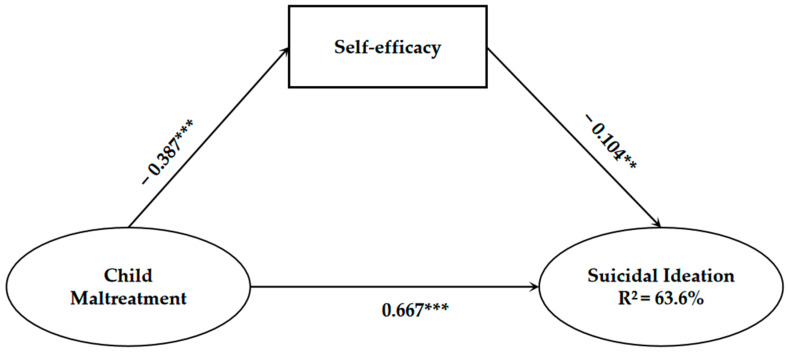
Results of structural model (*** *p* < 0.001; ** *p* < 0.01).

The results of the standardized path are shown in Figure 2. Regarding the relationship between maltreatment and suicidal ideation, after controlling for socio–demographic variables, maltreatment is positively correlated with suicidal ideation (β = 0.667, *p* < 0.001), indicating that the higher the level of maltreatment experienced by the left-behind children, the stronger the level of their suicidal ideation. After controlling for socio–demographic variables, child maltreatment also has an indirect effect on suicidal ideation through the mediating effect of self-efficacy. Specifically, maltreatment shows a significantly negative relationship with self-efficacy (β= −0.387, *p* < 0.001), indicating that the higher the level of maltreatment, the lower the level of self-efficacy among left-behind children. Meanwhile, Self-efficacy shows a significantly negative correlation with the suicidal ideation of left-behind children (β = −0.104, *p* < 0.01), indicating that the lower level of self-efficacy, the stronger the level of their suicidal ideation. In conclusion, maltreatment not only has a direct effect on the suicidal ideation of left-behind children, but also can have an indirect effect through the partially mediating effect of self-efficacy.

Among all the control variables, gender (β = 0.178, *p* < 0.001), household registration (β = 0.190, *p* < 0.001), and single-parent status (β = 0.080, *p* < 0.05) have significant effects on left-behind children’s suicidal ideation. Specifically, the female left-behind children have stronger suicidal ideation compared to the males. The left-behind children with urban household registration have stronger suicidal ideation than the rural ones. Left-behind children living in single-parent families have higher levels of suicidal ideation. This study also finds that being an only child has no significant effect on suicidal ideation. The path coefficients of the structural model are shown in Table 4.

### 4.5. Gender Differences

To determine whether the path coefficients from maltreatment to suicidal ideation varied significantly between males and females, multi-group analysis was employed. We contrasted the unconstrained model with the constrained model, in which the structural paths of males and females were determined to be equal. The two models’ significant chi-square differences (*p* < 0.001) revealed that there were gender differences. We adopted the critical ratios for differences (CRD) to identify the path differences. The results demonstrated that the path from maltreatment to suicidal ideation (CRD = 5.39, *p* < 0.001) was statistically different between male and female groups. Thus, gender moderated the impact of maltreatment on left-behind children’s suicidal ideation. The results also suggested that the impact of maltreatment on suicidal ideation was stronger for female left-behind children (β = 0.697, *p* < 0.001) than male left-behind children (β = 0.598, *p* < 0.001). These results suggest that there is a significant gender difference in the direct effect of maltreatment on left-behind children’s suicidal ideation, with females experiencing stronger influence than males.

## 5. Discussion

Using a 1355 left-behind children sample, this study investigates the association between child maltreatment and suicidal ideation, as well as the mediating effect of self-efficacy and the gender difference. The results demonstrate that child maltreatment significantly affects left-behind children’s suicidal ideation, and self-efficacy may mediate the relationship. Furthermore, the gender difference of the main effect is also suggested. Empirical data supports all research hypotheses in our study.

The findings support H1, indicating that a positive correlation exists between child maltreatment and suicidal ideation. Left-behind children in rural areas experience more physical and emotional abuse and neglect as a result of being separated from their parents. The prolonged abuse and neglect may create suicidal ideation in their daily lives [24,25]. The results support the findings of previous studies, suggesting that the influencing mechanism has the good validity and reliability across cultural contexts. Child maltreatment has a substantial direct impact on suicidal ideation, even in the left-behind children sample in rural China. Moreover, the findings are consistent with the integrated motivational volitional model which verified its cross-cultural adaptation in the Chinese context [15]. Childhood maltreatment, as negative life events, can predict individuals’ suicidal ideation and behavior [52]. The possible explanation is that although parents are aware of the importance of face-to-face care and emotional interaction in children’s development, their separation increases the difficulty of their care and communication for left-behind children. The left-behind children lack interpersonal interaction and emotional attachment with their parents, making them more vulnerable to physical and emotional abuse and neglect from their parents, which can increase the risk of suicidal ideation [9]. Due to financial constraints, caregiving pressures, and harsh parenting, left-behind children are also subject to physical and emotional neglect and abuse by their grandparents [10]. Maltreatment experiences may negatively affect their self-esteem, resilience, emotions, and socialization, which in turn can lead to deviant behaviors such as aggression, as well as suicidal ideation and attempts [4]. In addition, left-behind children may suffer from social rejection and maltreatment from their family members, teachers, and community members [11]. They may experience self-doubt, self-denial, and a loss of meaning in life, all of which can lead to suicidal ideation [17,18]. Consequently, when attempting to reduce suicidal ideation among left-behind children, counseling staff and social workers must consider the possibility of exposure to physical and emotional abuse and neglect, as well as sexual abuse.

The results reveal that child maltreatment is related to self-efficacy, which is in turn correlated with suicidal ideation. These findings also support H2, which demonstrate that child maltreatment significantly affects suicidal ideation through the mediating role of self-efficacy. Due to being separated from their parents, the left-behind children’s family generates a structure of division and dysfunction [53]. Prolonged lack of parental care and support causes left-behind children to suffer from physical, emotional, and psychological problems in varying degrees [54]. Living in an abusive situation can shape or alter left-behind children’s inner and outer perceptions, which may correlate with deficits in social cognition and self-efficacy [37,38]. Our findings are consistent with previous conclusions and suggest that childhood maltreatment is negatively correlated with self-efficacy in the left-behind children groups [39,40]. This study also verifies the association between self-efficacy and left-behind children’s suicidal ideation. Maltreatment impedes the construction of self-efficacy among left-behind children in rural areas, and then increases the possibility of generating suicidal ideation. In the face of adversity, these children tend to give up fighting their fates and develop a sense of meaninglessness, which leads to suicidal ideation [55]. This conclusion is consistent with findings from adolescents in Malaysia and South Carolina, indicating that low levels of self-efficacy increase suicidal ideation risks [44,56]. The findings of the mediating effect support the applicability of a developmental psychopathology perspective in explaining left-behind children groups in the Chinese context [27,31]. Thus, social workers should not only focus on the prevention of child maltreatment, but also pay more attention to the intervention of self-efficacy in left-behind children.

The findings also support H3 of the present study, suggesting that gender differences exist on the direct effect. Specifically, compared with left-behind boys, the effect of child maltreatment on suicidal ideation is stronger among left-behind girls. Thus, females are more vulnerable and are more likely to have suicidal ideation when exposed to child maltreatment. Previous studies in the US and UK indicated a strong correlation between maltreatment and suicidal ideation and suggested that maltreated females have a higher risk of suicidal ideation than males [46,47]. Our study confirms the previous findings by using a left-behind children sample in China. Meanwhile, these findings challenge the conclusion that a stronger relationship exists between maltreatment and suicidal ideation in boys in low-income nations than in girls [48]. Moreover, the significant gender differences in our study resolved controversies in the associations. Therefore, our study verifies the gender paradox in suicidal ideation, that is, that its incidence is higher among females than among males [48]. Several explanations exist for the results. First, owing to the rural preference for sons over daughters, daughters are in a relatively disadvantaged position, making them more vulnerable to exclusion, neglect, and abuse from grandparents. Thus, when left-behind daughters suffer maltreatment, the psychological burden and frustration cause them to have a high proclivity for suicidal ideation. Second, because most caregivers are preoccupied with their jobs, they are unable to provide more companionship and care to left-behind children. Compared to boys, girls are more emotionally sensitive and feel more loneliness and depression, which is one of the risk factors that can increase suicidal ideation. Third, parental absence leads to a higher probability that left-behind girls would be harassed and sexually abused by their caregivers and neighbors, which brings them serious psychological trauma and increases suicidal ideation. Thus, families, schools, and communities must endeavor to prevent and reduce maltreatment risks to weaken the negative effect on left-behind children, especially females.

## 6. Limitations

There are some limitations in this study. First, previous studies suggested that some other factors (e.g., self-control, resilience, social support) may mediate or moderate the relationship between maltreatment and suicidal ideation. Future research must incorporate these variables into the theoretical framework to investigate the indirect mechanism. Second, maltreatment was adopted as a latent variable in the present study. Thus, the different effects of each dimension of maltreatment need to be further explored. Third, given that we collected data in only one province in Mainland China, the results may not be applicable to other provinces. Consequently, further data collection in other regions must be conducted to test the generalization of the results.

## 7. Conclusions

Left-behind children are confronted with a range of psycho–social and developmental risks due to parental absence and childhood maltreatment. Our results unveiled that self-efficacy partially mediated the effect of maltreatment on suicidal ideation among left-behind children. Findings also revealed that the effect of maltreatment on left-behind children’s suicidal ideation was stronger for females than males. These findings suggest that social work interventions should focus on preventing childhood maltreatment or addressing its long-term negative effects on the psychological and behavioral development of left-behind children.

## Figures and Tables

**Table 1:** Descriptive statistics of socio–demographic characteristics (N = 1355).

	Frequency (N)	Percentage (%)
Gender		
Male	581	42.9
Female	774	57.1
Household registration		
Rural	600	44.3
Urban	755	55.7
Only child		
Yes	593	43.8
No	762	56.2
Single-parent status		
Yes	187	13.8
No	1168	86.2
Age	Mean = 14.99 (S.D. = 1.826)	

**Table 2:** Pearson correlations among key variables.

	Mean	S.D.	Maltreatment	Suicidal Ideation	Self-Efficacy
Maltreatment	1.500	0.468	-		
Suicidal ideation	0.415	0.213	0.565 **	-	
Self-efficacy	3.081	0.686	−0.352 **	−0.356 **	-

Note: ** *p* < 0.01 (2-tailed).

**Table 3:** Results of measurement model.

Latent Variables	Observed Variables	Factor Loading
Maltreatment	Emotional abuse	0.797 **
	Physical abuse	0.517 **
	Sexual abuse	0.431 **
	Emotional neglect	0.746 **
	Physical neglect	0.636 **
Suicidal ideation	Hopelessness	0.820 **
	Optimistic	0.776 **
	Sleep quality	0.509 **
	Hiding	0.464 **

Note. ** *p* < 0.01.

**Table 4:** Results of structural model.

Model Paths	B	β	SE	CR
Maltreatment→Suicidal ideation	0.300 ***	0.667	0.025	12.208
Maltreatment→Self-efficacy	−0.531 ***	−0.387	0.063	−8.395
Self-efficacy→Suicidal ideation	−0.034 **	−0.104	0.013	−2.611
Gender→Suicidal ideation	0.081 ***	0.178	0.016	4.993
Household registration→Suicidal ideation	0.024 ***	0.190	0.004	5.370
Only child→Suicidal ideation	0.015	0.034	0.016	0.964
Single parent family→Suicidal ideation	0.052 *	0.080	0.024	2.228

Note. (1) B: unstandardized path coefficient; β: standardized path coefficient; SE: standard error; CR = critical ratio; (2) *** *p* < 0.001; ** *p* < 0.01; * *p* < 0.05.

## Data Availability

The data presented in this study are available on request from the corresponding author.

## References

[1] Moody G., Cannings-John R., Hood K., Kemp A., Robling M. (2018). Establishing the international prevalence of self-reported child maltreatment: A systematic review by maltreatment type and gender. BMC Public Health..

[2] Manly J.T. (2005). Advances in research definitions of child maltreatment. Child Abus. Negl..

[3] Leitzke B.T., Pollak S.D., Deater-Deckard K., Panneton R. (2017). Child Maltreatment: Consequences, Mechanisms, and Implications for Parenting. Parental Stress and Early Child Development.

[4] Yang F., Shen Y., Nehring D. (2021). Maltreatment and depression among left-behind adolescents in rural China: The moderating roles of food security and depression literacy. Child Abus. Negl..

[5] Sidebotham P., Heron J. (2003). Child maltreatment in the “children of the nineties:” The role of the child. Child Abus. Negl..

[6] Pietrantonio A.M., Wright E., Gibson K.N., Alldred T., Jacobson D., Niec A. (2013). Mandatory reporting of child abuse and neglect: Crafting a positive process for health professionals and caregivers. Child Abus. Negl..

[7] Chan K.W. (2010). The household registration system and migrant labor in China: Notes on a debate. Popul. Dev. Rev..

[8] Chen M., Chan K.L. (2016). Parental absence, child victimization, and psychological well-being in rural China. Child Abus. Negl..

[9] Senaratne B., Perera H., Fonseka P. (2011). Mental health status and risk factors for mental health problems in left-behind children of women migrant workers in Sri Lanka. Ceylon Med. J..

[10] Zhang S., Xu Y., Hong J., Liu M., Liao M. (2022). Discrepancies between children’s and caregivers’ child maltreatment reporting and their associations with child wellbeing. Child Abus. Negl..

[11] Wang C., Tang J., Liu T. (2020). The sexual abuse and neglect of “left-behind” children in rural China. J. Child Sex. Abus..

[12] Xu T., Yue Q., Wang Y., Wang S., Liu W., Huang X. (2019). Perception on risk factors of child maltreatment in China: A qualitative study among health professionals. BMJ Open.

[13] Cao Y., Yang F. (2018). Self-efficacy and problem behaviors of school bully victims: Evidence from rural China. J. Child Fam. Stud..

[14] Duprey E.B., Handley E.D., Manly J.T., Cicchetti D., Toth S.L. (2021). Child maltreatment, recent stressful life events, and suicide ideation: A test of the stress sensitivity hypothesis. Child Abus. Negl..

[15] O’Connor R.C. (2011). The integrated motivational-volitional model of suicidal behavior. Crisis.

[16] Plunkett A., O’Toole B., Swanston H., Oates R.K., Shrimpton S., Parkinson P. (2001). Suicide risk following child sexual abuse. Ambul. Pediatr..

[17] Gong M., Zhang S., Li W., Wang W., Wu R., Guo L., Lu C. (2020). Association between childhood maltreatment and suicidal ideation and suicide attempts among Chinese adolescents: The moderating role of depressive symptoms. Int. J. Environ. Res. Public Health.

[18] Liu J., Fang Y., Gong J., Cui X., Meng T., Xiao B., He Y., Shen Y., Luo X. (2017). Associations between suicidal behavior and childhood abuse and neglect: A meta-analysis. J. Affect. Disord..

[19] Zelazny J., Melhem N., Porta G., Biernesser C., Keilp J.G., Mann J.J., Oquendo M.A., Stanley B., Brent D.A. (2019). Childhood maltreatment, neuropsychological function and suicidal behavior. J. Child Psychol. Psychiatry.

[20] Thompson M.P., Kingree J.B., Lamis D. (2018). Associations of adverse childhood experiences and suicidal behaviors in adulthood in a U.S. nationally representative sample. Child Care Health Dev..

[21] De Araújo R.M.F., Lara D.R. (2016). More than words: The association of childhood emotional abuse and suicidal behavior. Eur. Psychiatry.

[22] Schönfelder A., Rath D., Forkmann T., Paashaus L., Lucht L., Teismann T., Stengler K., Juckel G., Glaesmer H. (2021). Child abuse and suicidality in the context of the interpersonal psychological theory of suicide: A network analysis. Br. J. Clin. Psychol..

[23] Angelakis I., Gillespie E.L., Panagioti M. (2019). Childhood maltreatment and adult suicidality: A comprehensive systematic review with meta-analysis. Psychol. Med..

[24] Fellmeth G., Rose-Clarke K., Zhao C., Busert L.K., Zheng Y., Massazza A., Devakumar D. (2018). Health impacts of parental migration on left-behind children and adolescents: A systematic review and meta-analysis. Lancet.

[25] Zhang H., Liu M., Long H. (2021). Child Maltreatment and Suicide Ideation in Rural China: The Roles of Self-compassion and School Belonging. Child Adolesc. Soc Work J..

[26] Mossige S., Huang L., Straiton M., Roen K. (2016). Suicidal ideation and self-harm among youths in Norway: Associations with verbal, physical and sexual abuse. Child Fam. Soc. Work.

[27] Bandura A. (1977). Self-efficacy: Toward a unifyring theory of behavioral change. Psychol. Rev..

[28] Cicchetti D., Banny A., Lewis M., Rudolph K.D. (2014). A developmental psychopathology perspective on child maltreatment. Handbook of Developmental Psychopathology.

[29] Duprey E.B., Oshri A., Liu S. (2018). Childhood maltreatment, self-esteem, and suicidal ideation in a low-SES emerging adult sample: The moderating role of heart rate variability. Arch. Suicide Res..

[30] Locke T.F., Newcomb M.D. (2005). Psychosocial predictors and correlates of suicidality in teenage Latino males. Hisp. J. Behav. Sci..

[31] Cicchetti D. (2016). Socioemotional, personality, and biological development: Illustrations from a multilevel developmental psychopathology perspective on child maltreatment. Annu. Rev. Psychol..

[32] Harter S. (2012). The Construction of the Self: Developmental and Sociocultural Foundations.

[33] Maguire S.A., Williams B., Naughton A.M., Cowley L.E., Tempest V., Mann M.K., Teague M., Kemp A.M. (2015). A systematic review of the emotional, behavioural and cognitive features exhibited by school-aged children experiencing neglect or emotional abuse. Child Care Health Dev..

[34] Tubman J.G., Oshri A., Duprey E.B., Sutton T.E. (2021). Childhood maltreatment, psychiatric symptoms, and suicidal thoughts among adolescents receiving substance use treatment services. J. Adolesc..

[35] Oshri A., Carlson M.W., Kwon J.A., Zeichner A., Wickrama K.K.A.S. (2016). Developmental growth trajectories of self-Esteem in adolescence: Associations with child neglect and drug use and abuse in young adulthood. J. Youth Adolesc..

[36] Tirosh D., Tsamir P., Levenson E., Tabach M., Barkai R. (2013). Exploring young children’s self-efficacy beliefs related to mathematical and nonmathematical tasks performed in kindergarten: Abused and neglected children and their peers. Educ. Stud. Math..

[37] Feng J., Li S., Chen H. (2015). Impacts of stress, self-efficacy, and optimism on suicide ideation among rehabilitation patients with acute pesticide poisoning. PLoS ONE.

[38] Wu S.L., Yaacob S.N. (2017). Self-efficacy as a mediator of the relationship between parental closeness and suicidal ideation among Malaysian adolescents. Child Adolesc. Ment. Health.

[39] Yıldız Inanıcı S., Akgün B., Karataş H. (2019). Self-efficacy in abused and neglected pregnant women: Attachment theory and theory of mind perspectives. Aust. J. Forensic Sci..

[40] Singer M.J., Humphreys K.L., Lee S.S. (2016). Coping self-efficacy mediates the association between child abuse and ADHD in adulthood. J. Atten. Disord..

[41] Vaillancourt-Morel M.P., Bergeron S., Blais M., Hébert M. (2019). Longitudinal associations between childhood sexual abuse, silencing the self, and sexual self-efficacy in adolescents. Arch. Sex. Behav..

[42] Soffer N., Gilboa–Schechtman E., Shahar G. (2008). The relationship of childhood emotional abuse and neglect to depressive vulnerability and low self–efficacy. Int. J. Cogn. Ther..

[43] Abdel-Khalek A.M., Lester D. (2017). The association between religiosity, generalized self-efficacy, mental health, and happiness in Arab college students. Personal. Individ. Differ..

[44] Valois R.F., Zullig K.J., Hunter A.A. (2015). Association between adolescent suicide ideation, suicide attempts and emotional self-efficacy. J. Child Fam. Stud..

[45] Durand G., Velozo J.C. (2018). The interplay of gender, parental behaviors, and child maltreatment in relation to psychopathic traits. Child Abus. Negl..

[46] Polanco-Roman L., Alvarez K., Corbeil T., Scorza P., Wall M., Gould M.S., Duarte C.S. (2021). Association of childhood adversities with suicide ideation and attempts in Puerto Rican young adults. JAMA Psychiatry.

[47] Bebbington P.E., Cooper C., Minot S., Brugha T.S., Jenkins R., Meltzer H., Dennis M. (2009). Suicide attempts, gender, and sexual abuse: Data from the 2000 British Psychiatric Morbidity Survey. Am. J. Psychiatry.

[48] Seff I., Stark L. (2019). A sex-disaggregated analysis of how emotional violence relates to suicide ideation in low-and middle-income countries. Child Abus. Negl..

[49] Bernstein D.P., Stein J.A., Newcomb M.D., Walker E., Pogge D., Ahluvalia T., Stokes J., Handelsman L., Medrano M., Desmond D. (2003). Development and validation of a brief screening version of the Childhood Trauma Questionnaire. Child Abus. Negl..

[50] Xia Z.Y., Wang D.B., Wu S.Q., Ye J.H. (2002). Primary development of self-rating idea of suicide scale. J. Clin. Psychiatry.

[51] Schwarzer R., Bäßler J., Kwiatek P., Schröder K., Zhang J.X. (1997). The assessment of optimistic self-beliefs: Comparison of the German, Spanish, and Chinese versions of the general self-efficacy scale. Appl. Psychol..

[52] Devries K.M., Mak J.Y.T., Child J.C., Falder G., Bacchus L.J., Astbury J., Watts C.H. (2014). Childhood sexual abuse and suicidal behavior: A meta-analysis. Pediatrics.

[53] Mu G.M., Hu Y. (2016). Living with Vulnerabilities and Opportunities in a Migration Context: Floating Children and Left-Behind Children in China.

[54] Liang W., Hou L., Chen W. (2008). Left-behind children in rural primary schools: The case of Sichuan province. Chin. Educ. Soc..

[55] Lefebvre R., Fallon B., Van Wert M., Filippelli J. (2017). Examining the Relationship between Economic Hardship and Child Maltreatment Using Data from the Ontario Incidence Study of Reported Child Abuse and Neglect-2013 (OIS-2013). Behav. Sci..

[56] Isaac V., Wu C.Y., McLachlan C.S., Lee M.B. (2018). Associations between health-related self-efficacy and suicidality. BMC Psychiatry.

